# Draft Genome Sequence of Pseudomonas oceani DSM 100277^T^, a Deep-Sea Bacterium

**DOI:** 10.1128/genomeA.00254-18

**Published:** 2018-04-12

**Authors:** Elena García-Valdés, Margarita Gomila, Magdalena Mulet, Jorge Lalucat

**Affiliations:** a Microbiologia, Departament de Biologia, Universitat de les Illes Balears, Palma de Mallorca Balearic Islands, Spain; b Institut Mediterrani d’Estudis Avançats (IMEDEA, CSIC-UIB), Palma de Mallorca, Spain

## Abstract

Pseudomonas oceani DSM 100277^T^ was isolated from deep seawater in the Okinawa Trough at 1390 m. P. oceani belongs to the Pseudomonas pertucinogena group. Here, we report the draft genome sequence of P. oceani, which has an estimated size of 4.1 Mb and exhibits 3,790 coding sequences, with a G+C content of 59.94 mol%.

## GENOME ANNOUNCEMENT

Pseudomonas oceani is a Gram-negative bacillus isolated from deep seawater (1). Pseudomonas oceani belongs to the Pseudomonas pertucinogena group (2). This group is comprised primarily of marine and aquatic strains. The taxonomically closest strains to P. oceani, P. aestusnigri (3, 4) and P. pachastrellae (5), are from marine isolates and are characterized by the smallest genomes in the Pseudomonas genus.

The whole-genome shotgun sequence of P. oceani was performed on an Illumina platform in a combination of 300-bp paired-end reads. The Newbler Assembler version 2.7 software package (Roche) was used for *de novo* genome assembly. The draft genome size is 4,155,021 bp and contains 64 contigs, with an average contig length of 62,983 kb, a median coverage depth of 70×, and an average G+C content of 59.94 mol%. Prediction and annotation of the genome was performed using the NCBI Prokaryotic Genome Annotation Pipeline (https://www.ncbi.nlm.nih.gov/genome/annotation_prok). Analysis and comparison of the functional annotation was done using the KEGG Automatic Annotation Server (KAAS) (6). The genome has 3,849 genes, with a total of 3,790 coding sequences, 52 tRNA sequences, and 1 rRNA sequence identiﬁed in the chromosome.

Flagellation and twitching motility genes have been found. Genes encoding type II and VI secretion systems were localized in the genome. Few metabolic characteristics are present in this group of species. The utilization of putrescine, a differentiating characteristic between P. oceani DSM 100277^T^ and P. pachastrellae CCUG 46540^T^ or P. aestusnigri CECT 8317^T^, is corroborated by the presence of the genes for transporters of mineral and organic ions, such as iron III, sulfate, molybdate, and putrescine. Other genes present are related to phosphate, phosphonate, and urea transport, together with those for transport of iron complex siderophores, microcin C, and zinc. The genes for the multidrug-resistance efflux pumps MdtABC and MexGHI-OpmD have been annotated. Glycolysis, Entner-Doudoroff, and pentose phosphate pathways for sugar assimilation are present. Genes encoding alkane 1-monooxygenase and rubredoxin are related to fatty acid degradation. Putative nitrate and nitrite reductase genes for the conversion of nitrate to ammonia have been annotated, but the original phenotypic test was given as negative (1). EnvZ-OmpR two-component signal transduction system genes involved in osmotic stress response are present, together with a gene encoding ectoine synthase.

P. oceani has been annotated for 11 transposases, 4 of them belonging to the IS*3* family. A cluster of 24 phage-related genes is present.

### Accession number(s).

This whole-genome shotgun project has been deposited at DDBJ/ENA/GenBank under the accession number PPSK00000000. The version described in this paper is version PPSK01000000.
